# Multi-Modal Learning-Based Equipment Fault Prediction in the Internet of Things

**DOI:** 10.3390/s22186722

**Published:** 2022-09-06

**Authors:** Xin Nan, Bo Zhang, Changyou Liu, Zhenwen Gui, Xiaoyan Yin

**Affiliations:** 1 School of Information Science and Technology, Northwest University, Xi’an 710127, China; 2 The 7th Research Institute of Electronics Technology Group Corporation, Guangzhou 510310, China

**Keywords:** Internet of Things, multi-modal learning, equipment fault prediction, convolutional neural network

## Abstract

The timely detection of equipment failure can effectively avoid industrial safety accidents. The existing equipment fault diagnosis methods based on single-mode signal not only have low accuracy, but also have the inherent risk of being misled by signal noise. In this paper, we reveal the possibility of using multi-modal monitoring data to improve the accuracy of equipment fault prediction. The main challenge of multi-modal data fusion is how to effectively fuse multi-modal data to improve the accuracy of fault prediction. We propose a multi-modal learning framework for fusion of low-quality monitoring data and high-quality monitoring data. In essence, low-quality monitoring data are used as a compensation for high-quality monitoring data. Firstly, the low-quality monitoring data is optimized, and then the features are extracted. At the same time, the high-quality monitoring data is dealt with by a low complexity convolutional neural network. Moreover, the robustness of the multi-modal learning algorithm is guaranteed by adding noise to the high-quality monitoring data. Finally, different dimensional features are projected into a common space to obtain accurate fault sample classification. Experimental results and performance analysis confirm the superiority of the proposed algorithm. Compared with the traditional feature concatenation method, the prediction accuracy of the proposed multi-modal learning algorithm can be improved by up to 7.42%.

## 1. Introduction

In order to ensure the normal operation of large equipment in the Internet of things, an efficient and accurate fault diagnosis and prediction method is urgently needed. Usually, sensors are deployed to monitor the running status of equipment in real time. For example, temperature and humidity sensors can be used to obtain the current operating environment of the equipment. These various types of sensors can describe the operating conditions of the same equipment from different angles. Combining the data from multiple sensors not only improves the accuracy of fault prediction, but also reduces the industrial safety accidents caused by the failure of a single sensor.

Currently, there are a few available equipment fault diagnosis methods based on single-mode data fusion [1,2,3]. However, different faults of equipment have different characteristics. Furthermore, due to the different types and deployment locations of sensors, the ability to capture fault characteristics is also different. Therefore, the data collected by sensors have different contributions to the accuracy of fault diagnosis and prediction. Accordingly, data that can make a high contribution to the accuracy of fault diagnosis and prediction is called high-quality monitoring data, otherwise it is called low-quality monitoring data. Low quality monitoring data is expected to help high quality monitoring data to further improve the accuracy of fault diagnosis and prediction. The data gathered by different sensors belong to different modes and should be usually extracted by different methods. Moreover, the expression of different pattern features is too different to be directly used in discriminant analysis. Therefore, how to fuse the monitoring data of different types of sensors in different locations faces great challenges.

In recent years, multi-modal feature fusion technology [4] has attracted extensive attention of scholars and industry. Multi-modal data analysis has become a hot topic. How to explore the potential relationship between different modes is the key to multi-modal data fusion. GMA (Generalized Multiview Analysis) [5] only considers the discriminant information in views, and ignores the discriminant information between views. MvDA (Multi-view Discriminant Analysis) [6] considers both inter view and intra view information, thus, MvDA has better multi-modal learning ability. Inspired by MvDA, we use the common space projection method based on MvDA to fuse multi-modal monitoring data.

In this paper, we propose a multi-mode learning framework to fuse different quality monitoring data collected by diverse sensors. To balance the accuracy and cost of prediction, we use different methods to handle the data with different modes. Firstly, wavelet transform is used to transform low-quality monitoring data, and then their features are extracted. At the same time, one-dimensional convolutional neural network is used to extract the features of high-quality monitoring data, and noise is added to high-quality monitoring data to improve the robustness of the system, so as to conquer system noise and unknown interference. Finally, different features are mapped to the common space through the fusion model, the global optimal solution is obtained by using the trace difference method based on iteration, and the accurate prediction of equipment failure is achieve.

Our contributions are summarized as follows.

- We make the first attempt to launch an multi-modal learning algorithm on equipment fault prediction in the Internet of Things.
- We design a series of strategies to improve the accuracy of equipment fault prediction, use different methods to deal with different quality of detection data, and improve the robustness of fault prediction algorithm by adding noise to high-quality monitoring data.
- We evaluate the performance of our proposed multi-modal learning algorithm on the CWRU bearing dataset. Compared with the traditional feature concatenation method, the prediction accuracy of the proposed multi-modal learning algorithm can be improved by up to 7.42%.

The remainder of this paper is organized as follows. We survey the related works in Section 2. We introduce system model and the target problem in details in Section 3. The equipment fault prediction framework is described in Section 4. Experiment results and performance analysis are presented in Section 5. Finally, we conclude this paper in Section 6.

## 2. Related Work

In recent years, there has been a great deal of related work on equipment fault diagnosis and prediction. These existing research can be divided into three categories in terms of the involved technology.

Physical model-based equipment fault diagnosis schemes explore the relationship between input and output, and then establish a mathematical model to simulate the operation of the equipment. Hmida et al. [7] proposed a robust fault diagnosis method based on third-order Kalman filter. A real-time linear fault diagnosis system was constructed in [8]. Shah et al. [9] built the aerodynamic difference simulation of the normal state and fault state of the bearing. Benmoussa et al. [10] obtained the structural conditions of fault detectability and isolation based on the bond graph model, and finally realized the electromechanical system application of autonomous vehicle. James et al. [1] studied the fault diagnosis of automotive power system based on fault tree analysis. Jaise et al. [11] proposed a fault tree strategy for vehicle system fault diagnosis based on directed graph model. Physical model-based equipment fault diagnosis has high accuracy and high system stability, but professional knowledge is the key to establish the corresponding accurate model.

Signal analysis-based fault diagnosis methods judge whether the equipment fails by analyzing the time-frequency domain information of the sensor signal. Taking AR model, variational modal analysis and random forest classifier into account, Han [12] realized fault diagnosis of non-stationary features such as bearing vibration information. Borghesani et al. [13] improved the diagnostic accuracy of bearing inverse spectrum under variable speed. Bhakta et al. [2] extracted fault features using cepstrum, and then improved the accuracy of equipment fault diagnosis based on gradient boost algorithm. Coconcelli et al. [14] used an encoder to segment bearing signals in terms of speeds, and utilized short-time Fourier transform to diagnose faults. Wang et al. [15] enhanced the fault signal by using time-frequency reduction and short-time Fourier transform, so as to improved the performance of bearing fault diagnosis finally. Chen et al. [16] extracted time-frequency domain features based on short-time Fourier transform, and then improved the accuracy of fault classification by compressing the feature scale. Although these methods based on signal analysis have achieved good performance in equipment fault diagnosis, they highly rely on professional knowledge and need to extract different features for different equipment operating conditions.

Machine learning-based prediction method has been widely used in the field of equipment fault diagnosis and prediction because of its powerful feature extraction and processing ability. Kumar et al. [17] identified the fault state of pump by solving the optimal parameter problem of support vector machine. Ali et al. [18] quickly distinguished short-circuit faults under different frequencies by clustering electrical and mechanical faults based on the frequency response under different states. Kim et al. [19] analyzed the operation data of power plant by using statistical methods and clustering algorithm, and then proposed a neural network fault prediction model, which can predict the pipeline leakage fault. Sohaib et al. [3] set up a boiler pipe leakage fault detection model based on wavelet transform and deep neural network. Combined multi-scale convolution with data enhancement, Zhuang et al. [20] implemented the classification and corresponding severity of bearing faults. Qian et al. [21] proposed an adaptive superimposed convolutional neural network to solve the translation error and the boundary problem of CNN, and finally realized the classification of bearing faults over s small set of data samples. Although classical machine learning algorithms shows its superiority in fault diagnosis and prediction, system parameters need to manually be selected carefully. Furthermore, the functions suitable for the network also need to be chosen wisely. Deep neural network has become the key technology of fault diagnosis and prediction since 2006 because of its strong ability of feature extraction and model transfer.

## 3. System Model and the Target Problem

### 3.1. System Model

We consider an equipment operating state monitoring system composed of *J* different types of sensors, which are deployed in different locations of the equipment to collect information of different device components. Each sensor collects *K* samples. The equipment has *I* different faults. The feature extracted from the sensor data using CNN architecture is expressed as $D_{ijk}=\left(d_{ijk1},d_{ijk2},\ldots ,d_{ijkz}\right)$, i=1,2,…,I, j=1,2,…,J, k=1,2,…,K, *z* is the number of features extracted. Compared with the normal operation state, the data collected by the sensor will deviate from the reference value when a specific fault occurs. The performance characteristics of each fault can be captured by the deployed sensors to a certain extent. Furthermore, the same fault has different effects on different sensors, and different faults have different effects on the same sensor. Therefore, based on multi-modal learning of the data collected by all deployed sensors, we can diagnose equipment faults and accurately predict impending faults.

Due to the different characteristics of faults and the different deployment positions of sensors, the data collected by each sensor makes different contributions to the accuracy of fault diagnosis and prediction. To balance the performance and cost, for the monitoring data with high accuracy of fault diagnosis and prediction, we use a quite simple machine learning algorithm to learn its feature, and for the sensor data with low accuracy of fault diagnosis and prediction, we utilize a relatively complex machine learning algorithm to extract its features.

### 3.2. The Target Problem

#### 3.2.1. Pre-Experiments and Our Observations

**Figure 1:**
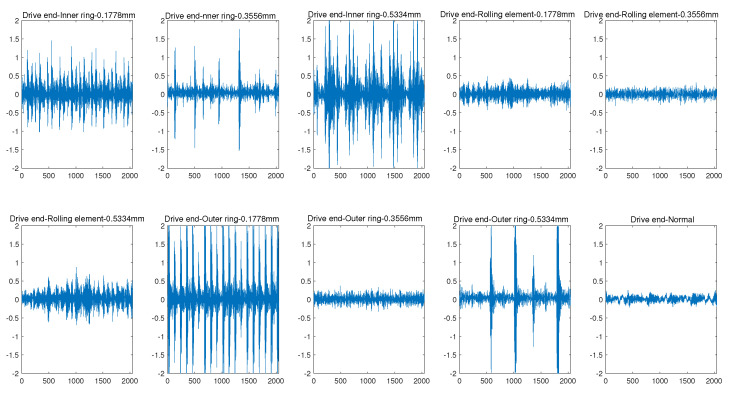
Time frequency diagram of failure samples at drive end.

**Figure 2:**
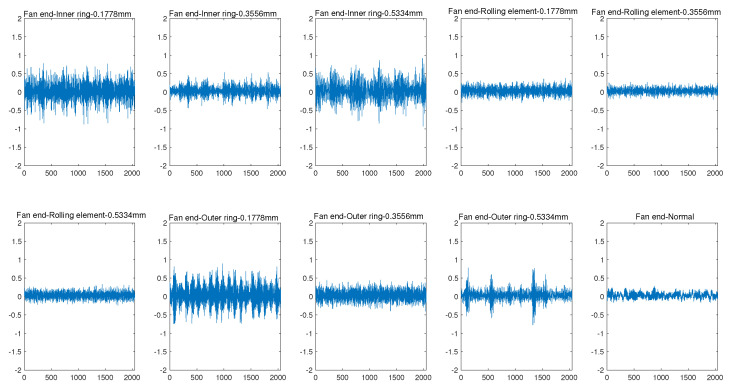
Time frequency diagram of failure samples at fan end.

We conduct pre-experiments on the CWRU bearing dataset, which is collected from a bearing platform. The bearing platform is mainly composed of motor, torque sensor, power detector and electronic control valve. The operating state of the bearing is monitored constantly with a vibration sensor at the drive end and a vibration sensor at the fan end. The sampling frequency of two sensors is 12 kHz. The failure of the bearing platform includes inner ring failure, outer ring failure and rolling element failure. Each type of fault corresponds to three different failure damage lengths, i.e., 0.1778 mm, 0.3556 mm and 0.5334 mm. Thus, there are nine fault states in total, as shown in Figure 1 and Figure 2. The first three subgraphs of Figure 1 are the inner ring faults with three corresponding different damage lengths at driver end, and so on, the last subgraph shows the normal operation state without any fault, which can be used as a benchmark.

**Figure 3:**
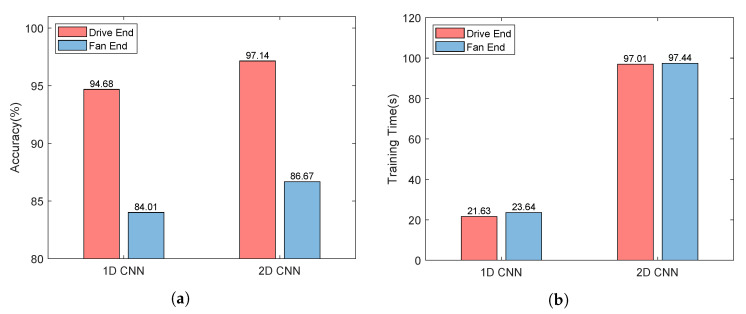
Accuracy and training time comparison of different CNN structures.

Inspiring by [22], we adopt a similar convolution network structure (called 2D CNN Structure) to extract features for monitoring data, and the settings of the 2D CNN Structure is shown in Table 1. Comparatively, we use the 1D CNN similar to the structure proposed in [23] to learn the characteristics of one-dimensional signals, and its settings is shown in Table 2. We made the following observations:

- As shown in Figure 3a, the monitoring data collected by the sensor at the drive end can always provide better performance whether 1D CNN or 2D CNN is used. Using 1D CNN, the prediction accuracy of fault prediction based on driver data can be improved by up to 10.67%. The evidence reinforces that the data collected by different sensors have different contributions to the accuracy of fault diagnosis and prediction. Thus, we distinguish between low-quality monitoring data and high-quality monitoring data.
- As shown in Figure 3a, for each type of monitoring data, compared with 1D CNN, 2D CNN can provide better performance thanks to its better learning ability. Using 2D CNN, the accuracy of fault prediction based on driver data can be improved by 2.46%, and the accuracy of fault prediction based on fan data can be improved by 2.66%. Combined with Figure 3b, the accuracy of fault prediction is improved by about 2.5%, while the training time increases by more than three times. Using 2D CNN, the training time based on driver data increases from 21.63 s to 97.01 s, and the training time based on driver data is increased from 23.64 s to 97.44 s. Therefore, it is necessary to tradeoff the prediction accuracy and the running time.
- The 2D CNN does help improve the accuracy of fault prediction. As shown in Figure 3a, the accuracy of using 2D CNN for two types of sensor data is higher than that of using 1D CNN. For the monitoring data collected by the sensor at the driver end, the prediction accuracy can reach 94.68% using ID CNN. For the monitoring data collected by the fan end sensor, even if 2D CNN is used, the prediction accuracy is only 86.67%. It can be seen that the monitoring data collected by each sensor has different contributions to the performance of fault prediction. Moreover, the performance of the monitoring data collected by the fan end sensor in fault prediction is not satisfactory, thus, its processing technology needs to be further optimized.

#### 3.2.2. The Target Problem

Generally, a variety of sensors are deployed to collect the operating status of the equipment from different angles and different granularity in an industrial Internet equipment fault diagnosis and prediction system. Because the sensor is deployed in different positions of important parts of the equipment, the distance to the equipment fault point is different, plus the influence of noise, resulting in the sensor providing different quality monitoring data. To improve the accuracy of fault diagnosis and prediction, multi-modal data fusion must be carried out on the monitoring data provided by all sensors.

Our goal is to design a high accuracy multi-modal learning algorithm for equipment fault prediction. In view of the different quality of various sensor data and their different contributions to the accuracy of equipment fault diagnosis and prediction, we need to tradeoff the cost and performance when designing multi-modal learning algorithms. Therefore, we should use a low complexity learning algorithm for high-quality monitoring data and a high-performance learning algorithm for low-quality monitoring data. Moreover, to improve the prediction accuracy of low-quality monitoring data, it is necessary to optimize the low-quality data and improve the accuracy of equipment fault prediction based on low-quality data as much as possible. In addition, due to the noise and unknown interference factors in the environment of industrial Internet, multi-modal learning algorithm needs to be highly robust.

## 4. The Proposed Equipment Fault Prediction Framework

**Figure 4:**
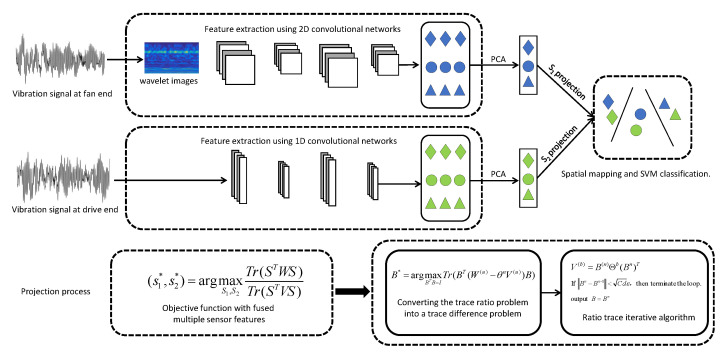
The framework of our proposed approach.

Corresponding to our target problem stated in Section 3.2.2, the proposed equipment fault prediction framework based on multi-modal learning adopts the following strategies: (1) to improve the performance of equipment fault prediction algorithm, the monitoring data are optimized first, i.e., the original monitoring data are resampled; (2) to tradeoff performance and cost, 1D CNN is used to extract the features of high-quality sensor data, e.g., the vibration signal at driver end collected by the sensor, and 2D CNN is utilized to learn the features of low-quality monitoring data, e.g., the vibration signal at fan end gathered by the sensor; (3) to improve the quality of low-quality monitoring data, wavelet analysis technology is used to analyze the low-quality information, reduce noise, and then reconstruct the wavelet time-frequency map; (4) to improve robustness of the fault prediction algorithm, noise is added to the high-quality monitoring data; (5) The features extracted by 1D CNN and 2D CNN are projected into the same common space using multi-modal data fusion. The spatial optimization problem is solved by the classic ratio trace iterative algorithm, and the fault classification is realized by support vector machine (SVM). The system framework is shown in Figure 4.

We summarize the key notations in Table 3.

### 4.1. Preprocessing and Feature Extraction

#### 4.1.1. Preprocessing

In order to improve the accuracy of fault diagnosis and prediction, we use resampling and noise adding methods to preprocess the data.

**Figure 5:**
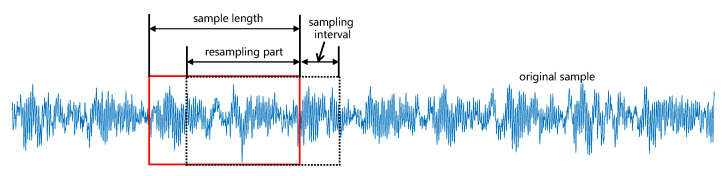
Illustration of the resampling method.

**Resampling.** It is worth noting that each type of fault sample needs to be normalized before being input into the convolutional neural network and the selection of the size of each data sample is critical. Moreover, sometimes the number of samples in the dataset is insufficient. Therefore, we expand and standardize the samples by resampling. More specifically, we set the sampling window as 1024 sampling points (for this bearing data set, each initial data sample shows obvious periodicity, to ensure that the selected sample size can cover the period of bearing damage, each sample was set to include 1024 sampling points), and the sampling interval between two samples is 256 sampling points, as shown in Figure 5. In other words, the resampling contains 768 sampling points.

**Noise Adding.** To improve the robustness of multi-modal data fusion algorithm, we mimic system noise or unknown interference by adding noise to the high-quality samples based on the additive Gaussian noise. The quality of the signal is generally measured in terms of SNR (Signal-Noise Radio), which is defined as:

(1) $$\mathrm{SNB}=10\log_{10}\left(\frac{P_{signal}}{P_{noise}}\right)$$

where $P_{noise}$ is the power of noise, and $P_{signal}$ is the useful power of the signal, which can be approximated by the variance.

The noise to be added to each sample can be calculated as:

(2) $$o=\sqrt{p_{noise}}randn\left(length\left(1024\right)\right)$$

where randn(·) denotes the function that generates the standard noise, and length(·) represents the length of the signal. The signal after adding noise is the superimposition of the original signal and the noise.

#### 4.1.2. Feature Extraction

For high-quality monitoring data, we use 1D CNN to extract its features and obtain the corresponding feature matrix $D_{ijk}=\left(d_{ijk1},d_{ijk2},\ldots ,d_{ijkz}\right)$, where *i* is the amount of equipment faults, *j* is the total number of mounted sensors, *k* is the number of samples, and *z* is the number of extracted features. For the low-quality sensor monitoring data, we first convert the original monitoring data into wavelet time-frequency map, and then use 2D CNN to learn its feature matrix also represented as $D_{ijk}=\left(d_{ijk1},d_{ijk2},\ldots ,d_{ijkz}\right)$.

Due to the sparse features learned by the convolutional neural network and their huge dimension, which will reduce the performance of subsequent public space projection and increase the iteration time, principal component analysis (PCA) is utilized to deal with each feature matrix. More specifically, by using PCA, we project the feature matrix onto a set of mutually orthogonal matrices, then calculate the variance between the projected data, and evaluate the importance of the feature matrix according to the variance. We let $D_{ijk}^{N}$ denote the standardized matrix of $D_{ijk}$. The covariance matrix of $D_{ijk}^{N}$ can be calculated as follows:

(3) $$Cov\left(M,Y\right)=\frac{\sum_{i=1}^{n}\left(M_{i}-\bar{M}\right)\left(Y_{i}-\bar{Y}\right)}{K-1}$$

where *M* and *Y* are two variables, $\bar{M}$ represents the mean of *M*, and $\bar{Y}$ is the mean of *Y*. We first calculate the eigenvectors of $D_{ijk}^{N}$, and then generate matrix $D^{C}$ based on these eigenvectors. Finally, we can construct the principal component matrix.

#### 4.1.3. The Loss Function

We define a loss function based on cross entropy to learn all parameters for 1D CNN and 2D CNN. Mathematically, the loss function can be expressed as

(4) $$L_{log}\left(Y,P\right)=-\log Pr\left(Y|P\right)=-\frac{1}{N}\sum_{i=0}^{N-1}\sum_{k=0}^{K-1}y_{i,k}\log p_{i,k}$$

where *Y* is the set of true labels, *P* is the set of predicted labels, *N* is the number of samples, *K* is the number of categories, $y_{i,k}$ denotes the *k*th label value of the *i*th sample, and $p_{i,k}$ denotes the probability that the model predicts the *i*th sample as the *k*th category.

### 4.2. Multi-Modal Learning Algorithm

Multi-modal fusion learning is the core of our proposed framework. The framework aims at two modal features: low-quality monitoring data features and high-quality monitoring data features, which are extracted at different granularity through different convolutional neural networks. Usually, these two features can predict equipment fault independently. To guarantee accuracy of the equipment fault prediction algorithm, we needs to fuse these two kinds of features before classification. Most of the feature fusion algorithms simply add and concatenate different features, and their performance are not satisfactory, because they ignore the correlation between high-quality features and low-quality features. Based on MvDA [6], we propose a supervised multi-modal learning algorithm for equipment fault prediction, which projects the features of high-quality monitoring data and low-quality monitoring data into a common space. Our learning objectives is to make samples of the same faults as close as possible in the common space, and samples of different types of faults as far away as possible in the common space.

For the aforementioned equipment fault monitoring system, we assume the multi-modal monitoring dataset $D=\left\{D_{ijk}\in R^{q_{j}}|i=1,2,\ldots ,I;j=1,2,\ldots ,J;k=1,2,\ldots ,k_{ij}\right\}$, where $d_{ijk}$ is the *k*th sample of the *j*th sensor in the *i*th fault, *I* is the number of fault types, *J* is the total number of sensors, $q_{j}$ is the feature dimension of sensor *j*, and $k_{ij}$ is the sample number of the *i*th fault of the *j*th sensor. At the same time, we let $F=\left\{F_{ijk}=s_{j}^{T}D_{ijk}|i=1,2,\ldots ,I;j=1,2,\ldots ,J;k=1,2,\ldots ,k_{ij}\right\}$ denote the projected samples, where $s_{j}$ is the projection matrix we need to learn. The optimal projection matrix can make the same fault samples as close as possible. Based on MvDA [6], mathematically speaking, the objective function of multi-modal learning algorithm can be expressed as follows:

(5) $$\left(s_{1}^{*},s_{2}^{*},\ldots ,s_{J}^{*}\right)=arg\max_{s_{1},s_{2},\ldots ,s_{J}}\frac{Tr(L_{out})}{Tr(L_{in})}$$

where $L_{in}$ is the within-class scatter matrix of projection in common space, $L_{out}$ expresses the between-class scatter matrix.

To simplify calculations, define $S=\left\{s_{1}^{T},s_{2}^{T},\ldots ,s_{J}^{T}\right\}$, $s_{j}$ is the l−th column of matrix *S*, the within-class scatter matrix $L_{in}$ and the between-class scatter matrix $L_{out}$ can be rewritten as:

(6) $$L_{in}=S^{T}VS$$

(7) $$L_{out}=S^{T}WS$$

where *V* and *W* are two block matrices, and their definitions are shown in MvDA [6].

By converting the objective function into the trace ratio problem, the solution of our multi-modal learning algorithm will be simplified. Thus, we rewrite Equation (5) as follows:

(8) $$\left(s_{1}^{*},s_{2}^{*},\ldots ,s_{J}^{*}\right)=arg\max_{s_{1},s_{2},\cdots ,s_{J}}\frac{Tr(S^{T}WS)}{Tr(S^{T}VS)}$$

The objective function represented by Equation (8) can be regarded as a trace ratio problem. It is difficult to obtain the global optimal solution of the multi-modal data fusion model by singular value decomposition. To solve this problem, an iterative algorithm based on ratio trace method is introduced to solve the trace ratio problem. Firstly, to maintain the solvability of the trace ratio problem, orthogonal constraints $S^{T}S=E$ are added, where *E* is the unit matrix.

We assume $\tilde{V}=W+V$. Equation (8) is updated as follows:

(9) $$S^{*}=arg\max_{S^{T}S=I}\frac{Tr(S^{T}WS)}{Tr(S^{T}\tilde{V}S)}$$

Given $0\leq \frac{Tr(S^{T}WS)}{Tr(S^{T}\tilde{V}S)}\leq 1$, since the multi-model learning algorithm preprocesses the monitoring data using PCA, $\tilde{V}$ is equal to 0 with negligible probability. Thus, $\tilde{V}$ can be converted as follows:

(10) $$\tilde{V}=B^{T}\Theta B$$

where $\Theta =\left[\theta_{1},\theta_{2},\ldots ,\theta_{c},\ldots ,\theta_{C}\right],\theta_{c}>0,c=1,2,\ldots ,C$, *C* is the number of positive singular values in $\tilde{V}$. Let S=QB, $Q\in R^{C\times d}$, *d* be the rank of *S*, the objective function can be updated as follows:

(11) $$S^{*}=arg\max_{B^{T}B=I}\frac{Tr(B^{T}W^{\left(u\right)}B)}{Tr(B^{T}V^{\left(u\right)}B)}$$

where $W^{\left(u\right)}=B^{T}WB,V^{\left(u\right)}=B^{T}\tilde{V}B$.

To solve the trace ratio problem of Equation (11) using singular value decomposition method, even if there is a closed solution, there may still be a large error with the optimal solution. Therefore, it is necessary to convert the trace ratio problem of Equation (11) into the trace difference problem. Thus, Equation (11) is converted as follows:

(12) $$B^{*}=arg\max_{B^{T}B=I}Tr\left(B^{T}\left(W^{\left(u\right)}-\theta^{n}V^{\left(u\right)}\right)B\right)$$

### 4.3. Global Optimum Solution Based on Eigenvalue Decomposition

Based on the eigenvalue decomposition method, the global optimal solution to the trace difference problem is achieved iteratively. The steps are described as follows:

- Initialize $B^{0}$ to any column orthogonal matrix.
- Calculate iteratively $\theta^{n}$ as follows:
  (13) $$\theta^{n}=\frac{Tr(B^{{n-1}^{T}}W^{\left(u\right)}B^{n-1})}{Tr(B^{{n-1}^{T}}V^{\left(u\right)}B^{n-1})}$$
- Construct the trace difference problem as follows:
  (14) $$B^{n}=arg\max_{B^{T}B=I}Tr\left(B^{T}\left(W^{\left(u\right)}-\theta^{n}V^{\left(u\right)}\right)B\right)$$
- Solve the trace difference problem based on eigenvalue decomposition method as follows:
  (15) $$\left(W^{\left(u\right)}-\theta^{n}V^{\left(u\right)}\right)b_{r}^{n}=\eta_{r}^{n}b_{r}^{n}$$
  where $\eta_{r}^{n}$ is the r−th largest eigenvalue of $\left(W^{\left(u\right)}-\lambda^{n}V^{\left(u\right)}\right)b_{r}^{n}$, $b_{r}^{n}$ is the corresponding eigenvector of $\eta_{r}^{n}$.
- Reconstruct the projection matrix to maintain orthogonality: Let $B^{n}=\left[b_{1}^{n},b_{2}^{n},b_{3}^{n},\cdots ,b_{d}^{n}\right]$, *d* is the rank of low-latitude feature, and perform singular value decomposition on $S^{\left(b\right)}=B^{n}{\left(B^{n}\right)}^{T}V^{\left(u\right)}B^{n}{\left(B^{n}\right)}^{T}$. The projection matrix can be updated as follows:
  (16) $$S^{\left(b\right)}=B^{n}\Theta^{b}{\left(B^{n}\right)}^{T}$$
- The termination criterion for iteration: if $\left|\right|B^{n}-B^{n-1}\left|\right|<\sqrt{Cd}\varepsilon$, Then the iteration ends, $B=B^{n}$.

The corresponding algorithm to find the global optimal solution to the trace difference problem is presented in Algorithm 1.
**Algorithm 1** Multi-modal Learning Algorithm1:**Input**: Two block matrices V and W.2:**Output**: The projection matrix B.3:Initialize $B^{0}$ to any column orthogonal matrix.4:**repeat**5:   Calculate $\theta^{n}$ using Equation (13).6:   Construct the trace difference problem based on Equation (14).7:   Solve the trace difference problem using Equation (15).8:   Reconstruct the projection matrix $B^{n}=\left[b_{1}^{n},b_{2}^{n},b_{3}^{n},\cdots ,b_{d}^{n}\right]$.9:   Perform singular value decomposition $S^{\left(b\right)}=B^{n}{\left(B^{n}\right)}^{T}V^{\left(u\right)}B^{n}{\left(B^{n}\right)}^{T}$.10:   Update the projection matrix using Equation (16).11:**until**$\left|\right|B^{n}-B^{n-1}\left|\right|<\sqrt{Cd}\varepsilon$.12:$B=B^{n}$.

## 5. Experiment Evaluation

We conduct experiments and analyze performance of the proposed algorithm on the CWRU bearing dataset, which is widely used for bearing fault diagnosis and prediction.

### 5.1. Dataset and Data Preprocessing

The CWRU bearing dataset is collected from a bearing platform. According to the monitoring system, the vibration sensors are mounted at the drive end and fan end of the bearing, and can provide high-quality monitoring samples (samples collected by the vibration sensor at drive end) and low-quality monitoring samples (samples gathered by the vibration sensor at fan end) at the same time. As mentioned in Section 3.2.1, the dataset includes 9 kinds of equipment fault samples and normal operation status data.

Because of the insufficient number of samples in the CWRU bearing dataset, we expand the data by resampling. Since each original sample contains 121,556 sampling points and shows obvious periodicity, according to the settings in Section 4.1.1, The expanded equipment fault samples of each type by resampling have 400 samples, we randomly selected 70% of them as the training set, and the remaining 30% as the testing set.

The accuracy of fault prediction based on low-quality samples is not satisfactory. If noise is added to low-quality samples, the performance of our multi-modal learning algorithm will be greatly degraded. To simulate noise or unknown system interference, we choose to add noise to high-quality samples, i.e., samples collected by the sensor at driver end.

**Figure 6:**
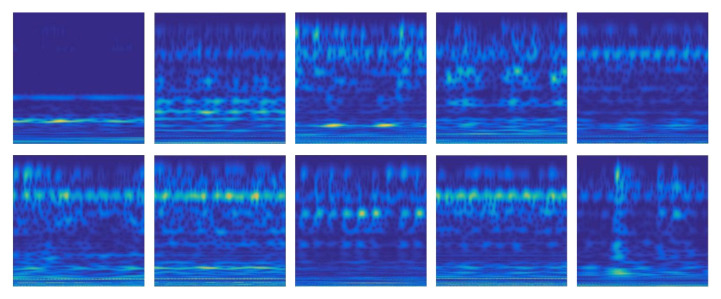
Time frequency diagram of fault samples after wavelet conversion.

To solve the problem that the prediction accuracy based on the fan end data is not high, we choose wavelet transform to optimize the low-quality samples from the sensor at fan end, and convert them into wavelet images. The converted wavelet images are shown in Figure 6. We compress the resolution of wavelet image to 64 × 64 and learn the features of wavelet image with 2D-CNN.

### 5.2. Baselines

We choose five algorithms that have achieved good performance in bearing fault prediction in recent years as benchmarks, including SDIAE [24] (Stacked Discriminat Information-based Auto-Encoder), VAEGAN-DRA [25] (Variational Autoencoding Generative Adversarial Networks with Deep Regret Analysis), SVM [26], SNN [27] (Spiking Neural Network), and SIRCNN [28] (Stacked Inverted Residual Convolution Neural Network).

### 5.3. Performance Analysis

**Prediction Accuracy Comparison.** As shown in Table 4, compared with the five benchmark algorithms, our proposed algorithm can achieve the highest fault prediction accuracy. After adding noise to high-quality samples, although the prediction accuracy of our algorithm decreases by 12.54%, the robustness of the algorithm has been enhanced. Moreover, the accuracy of the five benchmark algorithms in the driver side data is higher than that in the fan side data, which once again confirms that the samples have different quality and have different contributions to the accuracy of the prediction algorithm.

**Figure 7:**
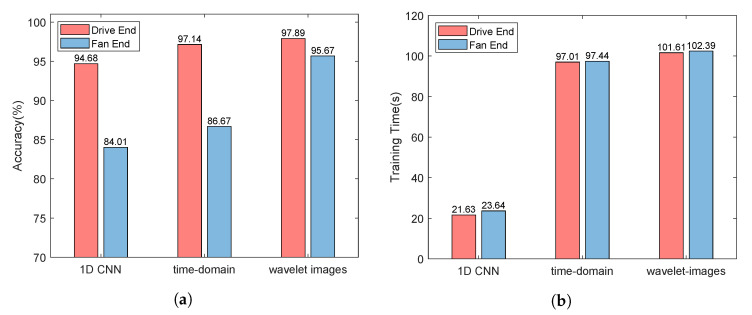
Accuracy and training time comparison.

**Effect of Wavelet Conversion** The results in Figure 7a confirm the effectiveness of wavelet transform in optimizing low-quality samples, and the accuracy of fault prediction based on fan end data is improved by 9%. At the same time, wavelet transform has little effect on improving high-quality samples, and the accuracy of fault prediction based on fan end data is only improved by 0.75%. It can be seen from Figure 7b that wavelet transform increases the time complexity of the algorithm within an acceptable range. Combined with Figure 7a,b, the training time is increased by 4.95 s, resulting in an improvement of 9% accuracy. In order to balance performance and cost, wavelet transform is only applicable to low-quality samples.

**Figure 8:**
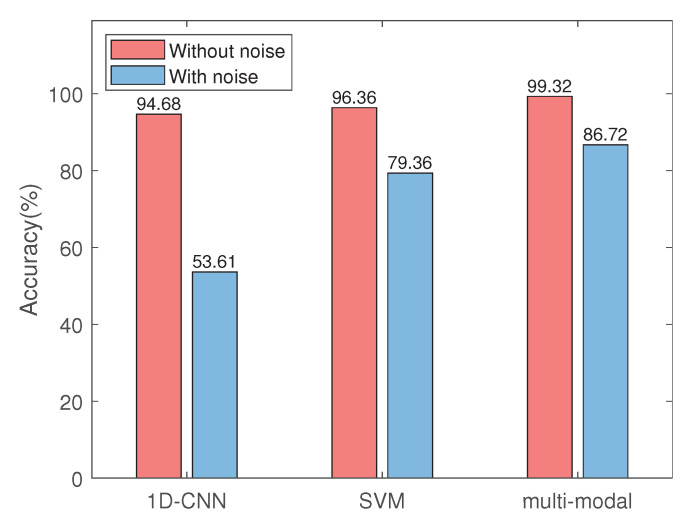
The influence Comparison of noise on accuracy.

**Effect of Noise.** 1D CNN implies that the features of data at the drive end is extracted directly; feature concatenation with SVM means that the features of data at drive end is extracted by 1D CNN, the feature of the wavelet images is extracted by 2D CNN, then two kinds of features are concatenated as input, SVM is used for classification finally; Multi-modal feature fusion refers to our proposed algorithm. As shown in Figure 8, Noise will reduce the prediction accuracy of the three methods, but the accuracy difference between with noise and without noise is different. ID CNN has the largest accuracy difference, and our method has the smallest accuracy difference, which proves that our algorithm has better robustness.

**Figure 9:**
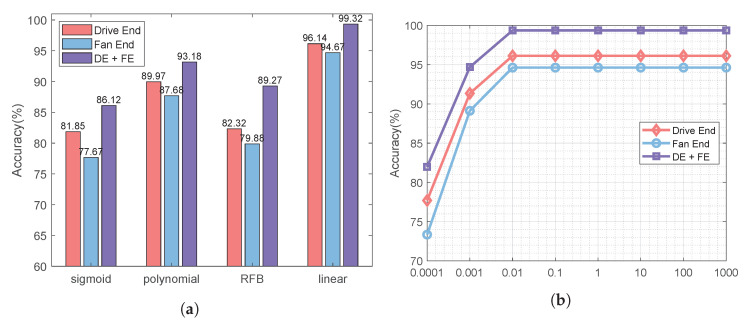
Effect of different parameters of SVM.

**Effect of SVM Parameters.** After projecting the sensor features into the common space, we use SVM as a classifier. Figure 9a shows the influence of four classical kernel functions on SVM. Obviously, the linear kernel has the best performance because of less parameters and faster speed. In Figure 9b, the influence of penalty factor on SVM is tested. The penalty factor becomes stable at 0.1, but changes little thereafter. Therefore, we set the penalty factor of SVM to 0.1. Moreover, we can also observe that the best performance can be obtained by fusing high-quality samples with low-quality samples. This proves that the fusion of low-quality samples is helpful to improve the accuracy of fault prediction.

**Figure 10:**
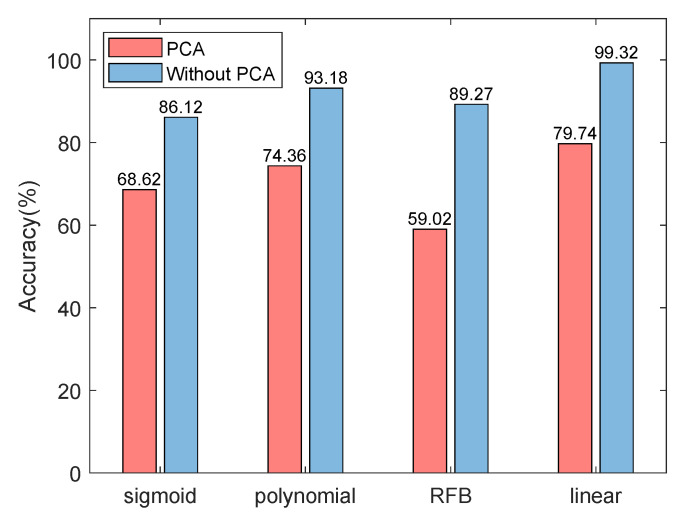
Accuracy comparison between using and not using PCA.

**Effect of PCA.** In the proposed framework, we used PCA to retain more than 95% of the principal components after each feature extraction. Figure 10 shows the influence of dimensionality reduction on the accuracy of fault prediction. The accuracy of fault prediction can be improved by up to 30% with PCA. Among the four kernel functions, PCA has the least influence on the sigmoid kernel function, and the accuracy is reduced by 17.5%.

## 6. Conclusions

This paper presents a multi-modal learning algorithm for equipment fault prediction. Taking the CWRU bearing dataset as an example dataset, the effectiveness of the following strategies is confirmed: (1) 1D CNN is enough to extract the features of high-quality monitoring data, and 2D CNN can extract the features of low-quality monitoring data converted by wavelet transform. (2) Adding noise to the high-quality monitoring data can improve the robustness of multi-modal learning algorithm. (3) Low quality monitoring data can be used as a compensation for high-quality monitoring data, which can improve the accuracy of equipment fault prediction. The core of the algorithm proposed in this paper is to better analyze the potential relationship between various modal data, and adopt different feature extraction methods for different quality monitoring data. The proposed model can provide useful guidance for the design of equipment fault monitoring system and the placement of sensors in the Internet of things, and allow a deeper exploration of effective methods of multi-modal monitoring data fusion in industrial Internet.

## Figures and Tables

**Table 1:** 2D CNN Structure.

Name of the Layers	Parameter Settings
1. Input layer	The size of the input image is 64 × 64 × 3, and perfroming *zerocenter* normalization.
2. Convolution layer	The number and size of convolution kernel are 8 and 5 × 5; step is [1,1]; filled as *same*.
3. Batch processing layer	batch normalization
4. Activation function layer	*Relu*
5. Pooling layer	max pooling; size is 2 × 2; setp is [2,2]; filled as 0.
6. Convolution layer	The number and size of convolution kernel are 16 and 5 × 5; step is [1,1]; filled as *same*.
7. Batch processing layer	batch normalization
8. Activation function layer	*Relu*
9. Pooling layer	max pooling; size is 2 × 2; setp is [2,2]; filled as 0.
10. Full connection layer	4096

**Table 2:** 1D CNN Structure.

Name of the Layers	Parameter Settings
1. Input layer	The size of the vibration signal is 1 × 1024, and perfroming *zerocenter* normalization.
2. Convolution layer	The number and size of convolution kernel are 8 and 1 × 1024; step is [1,1]; filled as *same*.
3. Pooling layer	max pooling; size is 1 × 512; filled as *same*.
4. Convolution layer	The number and size of convolution kernel are 16 and 1 × 512; step is [1,1]; filled as *same*.
5. Pooling layer	max pooling; size is 1 × 256; filled as *same*.
6. Full connection layer	4096

**Table 3:** Key notations.

Notation	Definition
*I*	the number of equipment failures
*J*	the number of types of sensors
*K*	the number of samples per sensor
*Z*	the number of features extracted
$D_{ijk}$	the feature extracted from the monitoring data
$P_{noise}$	the power of noise
$P_{signal}$	the useful power of the signal
*o*	the noise to be added to each sample
*D*	the multi-modal monitoring dataset
*F*	the projected samples
$L_{in}$	the within-class scatter matrix
$L_{out}$	the between-class scatter matrix
*S*	the set of sensor mapping matrix
W,V	block matrices
$q^{j}$	the feature dimension of the *j*-th sensor
*B*	the projection matrix

**Table 4:** Prediction accuracy comparison.

	SVM	SDIAE	VAEGAN-DRA	SNN	SIGCNN	OURS
Drive End	92.64%	97.83%	98.17%	98.23%	98.93%	99.32%
Fan End	83.97%	89.34%	86.07%	91.67%	93.93%	
Drive End (adding noise)	61.88%	71.94%	68.62%	82.62%	88.67%	86.78%

## Data Availability

The public dataset, CWRU (Case Western Reserve University Dataset), can be accessed by the following link: https://engineering.case.edu/bearingdatacenter/download-data-file.

## Abbreviations

In this study, we use the following abbreviations:

GMA	Generalized Multiview Analysis
MvDA	Multi-view Discriminant Analysis
CWRU	Case Western Reserve University
CNN	Linear dichroism
SVM	Support Vector Machine
SNR	Signal-Noise Radio
PCA	Principal Component Analysis
SDIAE	Stacked Discriminat Information-based Auto-Encoder
VAEGAN-DRA	Variational Autoencoding Generative Adversarial Networks with Deep Regret Analysis
SNN	Spiking Neural Network
SIRCNN	Stacked Inverted Residual Convolution Neural Network
